# Antipsychotic Drug Utilization and Adverse Drug Reaction Profiling in Patients With Schizophrenia at a Tertiary Care Hospital in Western India

**DOI:** 10.7759/cureus.23378

**Published:** 2022-03-22

**Authors:** Raakhi K Tripathi, Snehalata Gajbhiye, Sharmila Jalgaonkar, Nishtha Khatri, Mohsin Sayyed, Shubhangi Parkar

**Affiliations:** 1 Pharmacology and Therapeutics, Seth Gordhandas Sunderdas Medical College and King Edward Memorial Hospital, Mumbai, IND; 2 Psychiatry, Seth Gordhandas Sunderdas Medical College and King Edward Memorial Hospital, Mumbai, IND

**Keywords:** drug utilization, adverse drug reaction, antipsychotics, who indicators, cross-sectional

## Abstract

Introduction

Prescription pattern studies conducted in patients with schizophrenia have shown variability in the utilization of antipsychotics based on the geographical location of the study setting. Moreover, there is only a sparse number of studies specifically related to adverse drug reactions (ADRs) in schizophrenia. Hence, a need was felt to study the antipsychotic utilization pattern and adverse drug reactions in patients with schizophrenia in our setting.

Methods

This was a cross-sectional, observational study conducted at the psychiatry outpatient department (OPD) of a tertiary care hospital in India. Patients diagnosed to have schizophrenia as per the Diagnostic and Statistical Manual of Mental Disorders, Fifth Edition (DSM-5) were included in the study provided they had been prescribed antipsychotic medications at the study center for at least three months. The sociodemographic profile of the patients and caregivers was recorded, and prescription pattern assessment was done using WHO core drug use indicators. Information related to ADRs was recorded, and further assessment was done based on the causality, severity, and preventability of ADRs.

Results

A total of 250 patients were enrolled in the study. Risperidone (40.25%) and olanzapine (26.32%) were the most commonly prescribed antipsychotic drugs, while trihexyphenidyl was the most frequently prescribed concomitant medication. Among the 37 cases of adverse drug reactions that were recorded, amenorrhea, sedation, and weight gain were found to be the most common. The majority of ADRs were of mild severity in addition to being non-preventable.

Conclusion

It was observed that atypical antipsychotics were commonly prescribed in the study center, and the majority of the ADRs were mild and not preventable, which shows the adequacy of prescribing practices in the current setting.

## Introduction

Schizophrenia is a complex, chronic mental illness characterized by a variety of symptoms such as delusions, hallucinations, disorganized speech, catatonic behavior, and negative symptoms [1,2]. The median incidence of schizophrenia is 15.2 per 100,000 people. As per a systematic review that was conducted to assess the global burden of schizophrenia, the global age-standardized point prevalence of schizophrenia was estimated to be 0.28% in 2016, and prevalence rates did not seem to vary widely across different countries or regions. Population growth and aging are important factors that have led to a considerable and increasing burden attributable to schizophrenia, particularly in middle-income countries such as India [3].

In some parts of the world, there is a higher preference toward the usage of typical antipsychotics, while in other parts of the world, atypical antipsychotics are preferred [4]. A study pertaining to the drug use pattern of antipsychotics in schizophrenia conducted in the psychiatry outpatient department (OPD) of a tertiary care hospital in Andhra Pradesh showed that olanzapine (37.1%) was the most commonly prescribed antipsychotic [5], while another study conducted at a tertiary care center in New Delhi showed that risperidone (44.71%) was the most commonly prescribed antipsychotic [6]. One study executed in Mumbai showed that risperidone (21%) and quetiapine (20%) were the most commonly prescribed antipsychotics [7], while another study conducted in Karnataka showed that a combination of trifluoperazine and chlorpromazine was the most commonly prescribed (36%) in patients diagnosed with schizophrenia [8].

Moreover, as seen from past studies, there has been a considerable variation in antipsychotic drug use patterns in schizophrenia. Hence, prescription pattern studies are important in schizophrenia in order to ensure dynamic monitoring of the most commonly prescribed drugs in these patients. It is important to note that as far as schizophrenia is considered, no prescription pattern studies have been conducted in Western India over the last five years. Hence, the need was felt to conduct a study that evaluated prescription patterns in this region.

Studies in the past have shown that antipsychotic drugs can cause varied adverse drug reactions (ADRs) even at normal doses and can consequently lead to poor medication adherence, impaired quality of life, or fatality in extreme cases [9]. Knowledge among healthcare providers of the common ADRs due to antipsychotic drugs can help in the safe and rational use of antipsychotics and can also support early detection of ADRs [10,11]. In addition to this, we found that there were only a sparse number of studies that were specifically related to ADRs in schizophrenia.

Keeping the above findings in view, the current study was conceptualized to conduct a comprehensive evaluation of antipsychotic utilization pattern and adverse drug reactions in patients with schizophrenia.

## Materials and methods

The current study was a cross-sectional, observational study conducted in the psychiatry outpatient department of Seth Gordhandas Sunderdas Medical College and King Edward Memorial Hospital, Mumbai, India. The study was initiated after obtaining permission from the Institutional Ethics Committee (EC/103/2017), and the study was registered with the Clinical Trials Registry of India (CTRI/2018/03/012533). The study was conducted from January 2018 to June 2019.

Selection criteria

A purposive treatment-seeking sample of patients was recruited in the study after obtaining patient written informed consent. Patients who were aged between 18 and 65 years, diagnosed to have schizophrenia as per the Diagnostic and Statistical Manual of Mental Disorders, Fifth Edition (DSM-5) criteria, and prescribed antipsychotic medications at the study site for at least three months were included in the study. The exclusion criteria were newly diagnosed patients, treatment-naïve patients, patients with other psychiatric disorders/neurological disorders, patients not possessing the mental capacity to give consent, and critically ill patients.

Data collection form

A case record form (CRF) was designed to include information regarding patient demographic details such as age, sex, literacy level, socioeconomic status of the patients according to the modified Kuppuswamy scale [12], disease duration, and comorbid conditions. Details of the current prescription given to patients on the day of the interview as well as of prescriptions given over the last three months were recorded in the CRF. If there was any change in prescription, it was accounted for as a new prescription and was added to the total prescription count. The prescription details recorded in the CRF included generic name, brand name, dosage form, dose, frequency, and duration (days/months). Consultation time was also documented. Prescriptions were evaluated for completeness of data. If the prescriptions were found to be incomplete, a further assessment was done to gauge whether the incompleteness was in terms of dose, dosage form, frequency, duration (days/months), or dosage instructions, and this was noted in the CRF. The CRF was also designed to capture particulars of adverse event (AE) and adverse drug reactions (ADRs) experienced by the patients, if any, over the last three months.

Assessment tools

WHO drug use indicators (prescribing indicators, health facility indicators, and patient care indicators) were utilized for prescription pattern analysis [13]. The WHO-Uppsala Monitoring Centre (UMC) scale and Naranjo algorithm were used for causality assessment of ADRs [14]. The modified Hartwig and Siegel scale was used to determine ADR severity, while the modified Schumock and Thornton scales were used for the preventability assessment of ADRs [15,16].

Study procedure

All patients presenting to the psychiatry OPD with an assigned diagnosis of schizophrenia as per the DSM-5 by a trained psychiatrist were included, and written informed consent was taken. Details pertaining to patient sociodemographic factors, prescription patterns, and adverse drug reactions were recorded in the CRF.

Statistical analysis

The current study was conducted over a period of 18 months, and the sample size was based on the number of patients that could be enrolled over this duration. Data were entered in Excel, and descriptive statistics were used for this study. Data were expressed as absolute values and percentages.

## Results

A total of 250 patients were recruited in the study over a duration of 18 months. The majority of the patients (66.27%) were male, and the mean age of the patients was 40.44 ± 11.68 years. Details pertaining to sociodemographic profiles are shown in Table 1. The median disease duration of schizophrenia in the enrolled participants was 7 (IQR: 4-15) years. The common comorbid conditions were diabetes mellitus (n = 20), followed by hypertension (n = 11).

**Table 1:** Demographic data of the patients

Demographic profile		Number of patients (%) (n = 250)
Gender	Males	165 (66%); mean age: 40.85 ± 11.35 years
	Females	85 (34%); mean age: 39.65 ± 12.32 years
Socioeconomic status	Upper class (26–29)	2 (0.8%)
	Upper middle (16–25)	78 (31.2%)
	Lower middle (11–15)	109 (43.6%)
	Upper lower (5–10)	59 (23.6%)
	Lower (<5)	2 (0.8%)
Education status	Illiterate	8 (3.2%)
	Primary school education	18 (7.2%)
	Middle school education	57 (22.8%)
	High school education	78 (31.2%)
	Intermediate education or post-high school diploma	58 (23.2%)
	Graduate	30 (12%)
	Postgraduate or professional degree	1 (0.4%)

Prescription pattern

Overall, 323 prescriptions were obtained for analysis from the 250 patients that were enrolled in the study. The results of the WHO drug use indicators are summarized in Table 2.

**Table 2:** WHO drug use indicators

Prescribing indicators	Results
Average number of drugs per encounter	3.11 ± 1.27 (mean ± SD)
Average number of antipsychotics per encounter	1.44 ± 0.58 (mean ± SD)
Percentage of drugs prescribed by generic name	38.19%
Percentage of encounters with an injection prescribed	14.86%
Percentage of drugs prescribed from the national essential drugs list	58.36%
Percentage of drugs prescribed from the WHO essential drugs list	26.21%
Patient care indicators	Results
Average consultation time	4 minutes, 28 seconds
Health facility indicators	Results
Availability of a copy of the formulary list	Yes
Availability of key drugs	91.67%

The majority of the prescriptions, i.e., 286 (88.54%), out of 323 prescriptions, were complete. Among the incomplete prescriptions, the majority were incomplete in terms of dose (n = 31). The dose had been written for all antipsychotics. This incompleteness in terms of the dose was seen only in the case of concomitant medications. Dosage forms were mentioned in all prescriptions. The frequency of drug administration was not mentioned in one prescription, while the duration of treatment was not mentioned in two prescriptions. The designation/signature of the prescribing clinician was missing in three prescriptions. A total of 441 antipsychotics were prescribed in 323 prescriptions. Among the 441 antipsychotics, the majority, i.e., 319 (72.34%), were prescribed from the key drug list, while 122 (27.66%) were not from the key drug list. As per the WHO, key drugs refer to a short list of essential drugs that must always be available at the hospital formulary. It is a concise version of the hospital formulary list. In the current study, investigators obtained this list from the hospital dispensary and checked the list to compile the drugs that were used in psychiatric practice. The following drugs were included in the key drug list: amitriptyline, carbamazepine, diazepam, escitalopram, haloperidol, imipramine, lorazepam, olanzapine, sodium valproate, trifluoperazine, trihexyphenidyl, and risperidone.

Flupenthixol, clozapine, aripiprazole, amisulpride, quetiapine, chlorpromazine, ziprasidone, and zuclopenthixol were the drugs that were not from the key drug list. The utilization of the individual antipsychotics is depicted in Table 3. Four types of antipsychotic fixed-dose combinations (FDCs) were seen in the prescription pattern analysis. The FDC of risperidone + trihexyphenidyl (n = 19) was most commonly prescribed, followed by the FDCs of trifluoperazine + trihexyphenidyl (n = 4), olanzapine + fluoxetine (n = 1), and chlorpromazine trifluoperazine + trihexyphenidyl (n = 1). The prescribed daily dose/defined daily dose (PDD/DDD) ratio was found to be highest for haloperidol (2.5), while it was found to be lowest for quetiapine and chlorpromazine (0.25). This ratio was found to be 1 in the case of olanzapine. This is shown in Table 3.

**Table 3:** PDD/DDD ratio calculation for the prescribed antipsychotics ATC: Anatomical Therapeutic Chemical

Drug	ATC class	DDD (mg)	PDD/DDD	
			Median	IQR
Risperidone	N05AX08	5 mg	0.8	0.8–1.2
Olanzapine	N05AH03	10 mg	1	0.94–1.5
Trifluoperazine	N05AB06	20 mg	0.5	0.25–0.75
Haloperidol	N05AD01	8 mg	2.5	0.78–3.13
Clozapine	N05AH02	300 mg	0.33	0.17–0.5
Aripiprazole	N05AX12	15 mg	0.67	0.67–1
Amisulpride	N05AL05	400 mg	0.38	0.25–0.5
Quetiapine	N05AH04	400 mg	0.25	0.06–0.25
Chlorpromazine	N05AA01	300 mg	0.25	0.15–0.33
Ziprasidone	N05AE04	80 mg	0.4	-

Atypical antipsychotics were prescribed in 309 (95.67%) out of 323 encounters, while typical antipsychotics were prescribed in 158 (48.9%) out of 323 encounters. One antipsychotic was prescribed in 196 (60.68%) encounters, while more than one antipsychotic was prescribed in 127 (39.32%) encounters.

Anticholinergics were prescribed in 267/323 (82.66%) encounters, while anxiolytics were prescribed in 107/323 encounters, i.e., 33.13%, as shown in Table 4. The commonly prescribed drugs were anticholinergic - trihexyphenidyl (n = 241), anxiolytic - lorazepam (n = 62), antidepressant - fluoxetine (n = 8), and mood stabilizer - sodium valproate (n = 9).

**Table 4:** Utilization of the individual drugs acting on the CNS drugs (other than antipsychotics)

Drug class	Name of the drug	Number of encounters in which the drug was prescribed
Anticholinergics	Trihexyphenidyl	241
	Promethazine	26
Anxiolytics	Lorazepam	62
	Clonazepam	23
	Diazepam	15
	Chlordiazepoxide	7
Antidepressants	Fluoxetine	8
	Escitalopram	6
	Imipramine	4
	Sertraline	1
	Fluvoxamine	1
	Amitriptyline	1
Mood stabilizers	Sodium valproate	9
	Oxcarbazepine	1
	Lithium	1
	Divalproex	1
	Topiramate	1
Cholinesterase inhibitors	Donepezil	3

The other concomitant medications prescribed were multivitamin B complex (n = 56), ranitidine (n = 14), paracetamol (n = 13), pantoprazole (n = 12), calcium lactate (n = 7), ferrous sulphate + folic acid (n = 5), aluminum hydroxide + magnesium hydroxide (n = 3), and omeprazole (n = 2).

Change in prescription was seen in 73 encounters over the last three months. This change was due to a change in dose, frequency, or type of antipsychotic prescribed in 28 encounters, while in the remaining 45 encounters, the change in prescription was due to a change in the dose, frequency, or type of concomitant medications other than antipsychotics.

Adverse drug reactions

A total of 67 AEs were found in 61/250 (24.4%) patients. Asthenia (n = 7) was the most common AE, followed by sedation (n = 5). As per the WHO-UMC and Naranjo algorithm, 37/67 (55.22%) AEs were causally related to the treatment. These 37 ADRs were found in 33 (13.2%) patients. Details of the adverse drug reactions are depicted in Table 5.

A single ADR could be attributable to more than one drug if the patient was on multiple medications. Twenty-three ADRs were attributable to risperidone, nine ADRs to olanzapine, four ADRs to trifluoperazine, three ADRs to clozapine, and one ADR each to haloperidol and flupenthixol.

The type of ADR attributable to each antipsychotic is indicated in Table 5. ADRs such as tremors and tardive dyskinesias were primarily attributable to typical antipsychotics such as trifluoperazine and haloperidol, while other ADRs were attributable to atypical antipsychotics.

As per the WHO-UMC causality assessment scale, 31/37 ADRs were possibly related to the antipsychotic medication, while 6/37 ADRs were probably related. As per the Naranjo algorithm, 27/37 ADRs were possibly related to the antipsychotic medication, while 10 ADRs were probably related.

**Table 5:** Drugs implicated for each type of ADR

ADR detected	Number of ADRs (n = 37)	Implicated drug
Amenorrhea	4	Risperidone (n = 4)
Sedation	4	Risperidone (n = 2), clozapine (n = 1), olanzapine (n = 1)
Weight gain	4	Risperidone (n = 3), olanzapine (n = 1), clozapine (n = 1)
Dry mouth	3	Risperidone (n = 2), clozapine (n = 1), trihexyphenidyl (n = 2)
Somnolence	3	Risperidone (n = 3)
Tardive dyskinesia	3	Trifluoperazine (n = 3), olanzapine (n = 1)
Weakness	3	Risperidone (n = 3), flupenthixol (n = 1), trihexyphenidyl (n = 2)
Oligomenorrhoea	2	Risperidone (n = 2)
Constipation	1	Risperidone (n = 1)
Headache	1	Risperidone (n = 1)
Hyperglycemia	1	Olanzapine (n = 1)
Hyperhidrosis	1	Olanzapine (n = 1)
Hyperprolactinemia	1	Risperidone (n = 1)
Leucocytosis	1	Olanzapine (n = 1)
Paresthesia	1	Olanzapine (n = 1)
Seizure	1	Olanzapine (n = 1)
Sexual dysfunction	1	Risperidone (n = 1)
Tremors	1	Haloperidol (n = 1)
Throat irritation	1	Olanzapine (n = 1)

As per the modified Hartwig and Siegel scale, 30 ADRs were mild while 7 ADRs were moderate. As per the preventability assessment conducted by Schumock and Thornton Scale, 20 ADRs were not preventable, 16 ADRs were probably preventable, while 1 ADR was definitely preventable.

## Discussion

Prescription pattern studies conducted in patients with schizophrenia have shown variability in the utilization of antipsychotics based on the geographical location of the study setting. Studies conducted in the past have laid emphasis on the issue of ADRs related to antipsychotic treatment in schizophrenia. However, there is a paucity of data pertaining to ADRs caused by antipsychotics in the Indian context [6,17]. Taking this into consideration, we felt that there was a need to study the prescription patterns and adverse drug reactions in schizophrenia patients.

The average number of antipsychotics per encounter in our study was realized to be 1.44 ± 0.58. This number was found to be lesser compared to the study conducted by Nukala et al., in which this number was found to be 1.19 [18]. Surveys on prescribing practices in various regions of the world have identified the frequent and consistent use of more than one antipsychotic, with a prevalence as high as 50% in some clinical settings. Combination antipsychotics are commonly used to gain a greater and more rapid therapeutic response. However, current evidence on this topic is equivocal. Although not routinely recommended by guidelines, there are exceptional situations where the use of >1 antipsychotic can be justified [19].

The percentage of antipsychotics prescribed with generic names was analyzed to be 38.19%. In the study by Kumar et al., it was seen that only 16.22% of drugs were prescribed by generic name [6], while in the research done by Munjely et al., generic drug prescribing was found to be 73.4% [20]. This implies that the practice of prescribing drugs by generic names varies across different hospital setups. The only injectable drug that was prescribed was flupenthixol (14.8%), which is considered helpful in patient noncompliance [21].

The average consultation time per encounter was found to be four minutes and 29 seconds. A study by Elmore et al. found that there was no association between patient experience and consultation time, and patients may report good experiences even with a short consultation time. Even so, the authors of the above study suggested that longer consultations may be needed to achieve better clinical effectiveness and patient safety [22]. Prescribing information about all the antipsychotics was found to be complete. This could be justified by the fact that antipsychotics are supervised medications, and clinicians take extra precautions while prescribing the same.

The current study showed that atypical antipsychotics were the preferred choice of treatment in the majority of the patients. Evidence shows that there is no clinically significant difference in the efficacy of atypical and typical antipsychotics, but atypical antipsychotics are preferred by physicians since they have a better safety profile [23].

Risperidone was found to be the most commonly prescribed antipsychotic, followed by olanzapine and trifluoperazine. Our findings were in concordance with a survey conducted by Grover et al.; it was found that risperidone (30%) and olanzapine (30%) were the most preferred antipsychotics among the Indian psychiatrists [24]. In a study conducted by Shrivastava et al., risperidone (20.8%) was the most commonly prescribed antipsychotic, followed by quetiapine (19.8%). We saw similar findings in our study with respect to risperidone, but in our study, quetiapine was present in merely 4.33% of the encounters. However, Shrivastava et al. only looked into the encounters in which atypical antipsychotics were prescribed [7]. An important reason for the greater prescribing of risperidone and olanzapine was that these drugs were available free of cost in the hospital formulary.

The PDD/DDD ratio was found to be exactly 1 in the case of olanzapine, while the ratio was found to be deficient in the case of all other antipsychotics except for haloperidol, where the ratio was 2.5. Clinical practice guidelines indicate that Indian patients require lower doses of antipsychotic drugs compared to the Western population [25]. This could justify the deficit in the PDD/DDD ratio seen with the majority of antipsychotics in this study. The current finding could also be justified by the dose down titrations required in patients responding to antipsychotic treatment. For outpatients, the maintenance dose of antipsychotics is lower than that recommended for acute psychiatric treatment [26], while the high PDD/DDD ratio is seen with haloperidol, as it is a highly potent antipsychotic, generally prescribed to patients with symptoms of severe psychosis such as aggression and suicidal tendencies. It is in such situations that treatment guidelines must be viewed in the light of clinical judgment.

With respect to the FDCs containing antipsychotics, it was found that the various FDCs included that of risperidone + trihexyphenidyl (n = 19), trifluoperazine + trihexyphenidyl (n = 4), olanzapine + fluoxetine (n = 1), and chlorpromazine + trihexyphenidyl + trifluoperazine (n = 1). As per the report by Solanki et al., FDCs containing trihexyphenidyl with risperidone, trifluoperazine, chlorpromazine, or other antipsychotics cannot be considered as rational. The justification for its irrationality is based on the literature that the susceptibility for extrapyramidal side effects differs from person to person, and not all patients require the addition of trihexyphenidyl to overcome extrapyramidal side effects. Moreover, the antipsychotic-anticholinergic combination does not permit the use of need-based dose titration of trihexyphenidyl. This could be the cause of additional peripheral and central anticholinergic side effects in the patients, and the long-term use of trihexyphenidyl may cause cognitive impairment and tardive dyskinesia. The FDCs containing chlorpromazine and trifluoperazine could carry a risk of cumulative central nervous system toxicity and cardiac arrhythmias without any additional benefit. It is suggested that if need be, trihexyphenidyl should be separately prescribed alongside antipsychotics for patients that are more prone to develop extrapyramidal side effects and not as FDC [27].

The anticholinergic trihexyphenidyl (n = 241) was the most commonly prescribed co-medication, which was similar to the study conducted by Munjely et al. [20]. The current treatment guidelines do not recommend the use of anticholinergics prophylactically and also suggest the avoidance of its long-term use. In spite of this, the high use of long-term anticholinergic drugs with antipsychotics has been identified as an important issue requiring attention [28].

Among the 37 ADRs that were recorded, amenorrhea (n = 4), sedation (n = 4), and weight gain (n = 4) were the most common. In a study conducted by Chawla et al., it was found that decreased libido, weight gain, and menstrual irregularities were the most common ADRs occurring in patients on antipsychotics at a tertiary care hospital in Delhi [29].

In our study, the maximum percentage of ADRs was found to occur with risperidone (18%) and olanzapine (11%). Since risperidone and olanzapine were prescribed to larger number of patients, the chance of attributing ADRs to these highly prescribed drugs was higher. In a study conducted by Chawla et al., the results obtained were different. Chawla et al. showed that the ADRs were in almost 8% of the encounters in which risperidone and olanzapine were prescribed, and the majority of ADRs were seen with trifluoperazine (50% of the encounters in which it was prescribed). Extrapyramidal side effects are common with trifluoperazine, but in our setting, anticholinergics were prescribed in the majority of the prescriptions. This could be a reason why the percentage of encounters in which side effects were seen with trifluoperazine was just 5.26% in our study [29].

As per the WHO-UMC causality assessment scale, 31 ADRs were possibly related to antipsychotic medications, while six ADRs were probably related to antipsychotic medications. In a study conducted by Sengupta et al., it was found that tremors, weight gain, and constipation were the most commonly occurring ADRs in the psychiatric outpatient department in a tertiary care hospital in Kolkata [17]. Olanzapine was the drug that was found to be most commonly associated with ADRs in this study. Olanzapine was found to be causally related to 31.82% of the events.

The current study had a few limitations. A prescribed drug does not always mean that it was consumed by the patient, and hence, prescriptions do not provide a very accurate estimate of the drug utilization status. Nevertheless, this is a common limitation seen in cross-sectional drug utilization study designs.

## Conclusions

In conclusion, risperidone and olanzapine were the most commonly prescribed antipsychotic drugs, while trihexyphenidyl was the most frequently prescribed concomitant medication. The majority of the ADRs being “mild” and “not preventable” shows the adequacy of prescribing practices in the current setting. Psychiatrists and physicians must question patients about adverse drug reactions at every visit, and the importance of routine checkups and investigations must be reinforced among patients.
